# A novel scoring system for TIGIT expression in classic Hodgkin lymphoma

**DOI:** 10.1038/s41598-021-86655-8

**Published:** 2021-03-29

**Authors:** Ombretta Annibali, Antonella Bianchi, Alba Grifoni, Valeria Tomarchio, Mariantonietta Tafuri, Martina Verri, Giuseppe Avvisati, Anna Crescenzi

**Affiliations:** 1 Unit of Haematology, Stem Cell Transplantation, Campus Bio-Medico University Hospital, Álvaro del Portillo 200, 00128 Rome, Italy; 2 Unit of Pathology, Campus Bio-Medico University Hospital, Álvaro del Portillo 200, 00128 Rome, Italy; 3 Division of Vaccine Discovery, La Jolla Institute for Allergy and Immunology, 9420 Athena Circle, La Jolla, CA 92037 USA

**Keywords:** Cancer, Immunology

## Abstract

Clinical use of immune-checkpoints inhibitors (anti PD-1/PD-L1) resulted very effective for the treatment of relapsed/refractory classic Hodgkin Lymphoma (CHL). Recently, T cell Ig and ITIM domains (TIGIT) has been recognized as an immune checkpoint receptor able to negatively regulate T cell functions. Herein, we investigated the expression of TIGIT in CHL microenvironment in order to find a potential new target for inhibitor therapy. TIGIT, PD-1 and PD-L1 expression was evaluated in 34 consecutive patients with CHL. TIGIT expression in T lymphocytes surrounding Hodgkin Reed-Sternberg (HRS) cells was observed in 19/34 patients (56%), of which 11 (58%) had advanced stages. In 16/19 (84%) cases, TIGIT+ peritumoral T lymphocytes showed also PD-1 expression. All 15 TIGIT− patients had PD-L1 expression in HRS cells (100%) while among 19 TIGIT+ patients, 11 (58%) were PD-L1+ and 8 (42%) were PD-L1−. Using a new scoring system for TIGIT immunoreactivity, all TIGIT+ cases with higher score (4/19) were PD-L1−. Our results confirm co-expression of TIGIT and PD-1 in peritumoral T lymphocytes. Of relevance, we demonstrated a mutually exclusive expression of TIGIT and PD-L1 using new TIGIT scoring system able to identify this immunocheckpoints’ modulation. These results pave the way to new therapeutic strategies for relapsed/refractory CHL.

## Introduction

Classic Hodgkin lymphoma (CHL) has an unusual tumoral morphology in which the neoplastic population, characterized by large atypical cells, namely Hodgkin Reed-Sternberg (HRS) cells, is usually sparse and often surrounded by a non-neoplastic reactive immune cells background. Despite this reactive microenvironment, the lymphoma growth escapes host immune surveillance and the tumor, without therapy, is a life threatening disease. Routine therapeutic approach consists in poly-chemotherapy; however relapse and refractoriness may require different strategies^1^. To date, six immunocheckpoint inhibitors are approved for treatment of melanoma, lung cancer, head and neck cancer, bladder cancer and Merkel cell cancer and recently, the use of Programmed Death-1 (PD-1) inhibitor resulted very effective in relapsed or refractory Hodgkin lymphoma^2,3^. However, major clinical problems using these inhibitors are primary refractoriness and acquired resistance after a period of response, so deserving research for new antitumor immune activating agents^4^. In recent times, the novel co-inhibitory receptor T cell Ig and Immunoreceptor Tyrosine-based Inhibitory Motif, ITIM, domain (TIGIT) has displayed an important role in modulating immune responses in both autoimmunity mechanisms and cancer immune-escape. Several groups consistently reported that TIGIT expression was elevated on CD8+ tumor infiltrating lymphocytes (TILs) and regulatory T cell (T reg) in a variety of tumors. The expression of TIGIT is weakly or absent on naïve and resting T cells but can be induced by antigenic stimuli. TIGIT (also known as WUCAM, Vstm3, VSIG9), was first identified in 2009^5^; it is a member of the poliovirus receptor (PVR)/nectin family, and consists of three structural domains: an extracellular immunoglobulin variable-set (IgV) domain, a type 1 transmembrane domain, and an intra-cellular domain having both ITIM and an immunoglobulin tyrosine tail (ITT) region.

There are two prominent TIGIT ligands: CD155, originally identified as a poliovirus receptor (PVR) and CD112, also termed Nectin-2 or poliovirus receptor-related 2 protein (PVRL2). Both have been demonstrated overexpressed in various human malignancies, including colon cancer, lung adenocarcinoma, melanoma and pancreatic cancer^5^. By ligand interaction, TIGIT is able to inhibit lymphocytes activity through different mechanisms of action including modulation through its intracellular tail after binding to PVR, induction of PVR signaling in adjacent dendritic or tumor cells, and inhibition of CD226 signaling^6^.

The anti-TIGIT antibodies for the treatment of cancer and lymphoproliferative disease (tiragolumab) are currently under clinical trials and TIGIT might be attractive as a target for immunotherapy^3,5,7^.

Recently, TIGIT expression has been reported in CHL in lymphocytes surrounding HRS cells^8^. Although it was the first evidence of TIGIT expression in CHL, this result has not been correlated with other immunocheckpoints and a scoring system to grade TIGIT immunohistochemical expression has not been described.

Herein, we investigated the expression of TIGIT in CHL microenvironment in order to find a potential new target for inhibitor therapy. In agreement with the evaluation model for immunohistochemical expression of PD-L1 in tumors, we proposed a scoring system for TIGIT immunohistochemistry in CHL; moreover, we compared TIGIT results with PD-1 and PD-L1 expression to obtain a complete assessment of the immunomodulation of Hodgkin lymphoma microenvironment.

## Results

Study population included 34 patients with histologically proved CHL. The median age at diagnosis was 39 years (range 16–68), 17 were males and 17 females. Early stage was observed in 18/34 (53%) patients and 16/34 (47%) had advanced stage disease. Bulky disease was present in 7/34 (20%). Response to treatment was evaluated according to Cheson et al. criteria^9^. All patients were treated with ABVD (adriamycin, bleomycin, vinblastine, and dacarbazine) and out of 34 patients, 28 (82%) achieved a complete remission (CR), 2 (6%) a partial response and 4 (12%) presented a progressive disease. Relapse after response occurred in 3/30 (10%) responding patients. Table 1 summarizes clinical and biological characteristics at diagnosis of patients’ cohort (Table 1).

**Table 1:** Clinical and biological characteristics at diagnosis of patients’ cohort.

	Total	TIGIT Negative Score 0	TIGIT Positive Score 1–2–3
# Patients	34	15 (44%)	19 (56%)
Age median (range)	39 (16–68)	39 (18–52)	39 (21–68)
Sex (M/F)	17/17	5/10	12/7
**Stage**			
Early (I and II)	18 (53%)	10 (67%)	8 (42%)
Advanced (III and IV)	16 (47%)	5 (33%)	11 (58%)
Bulky yes	7 (20%)	3 (20%)	4 (21%)
**Response to treatment**			
CR	28 (82%)	11(73%)	17 (89%)
PR	2 (6%)	1 (6%)	1 (5%)
PD	4 (12%)	3 (20%)	1 (6%)
**Histology**			
NSCHL	17 (50%)	9 (53%)	8 (47%)
MCCHL	9 (26%)	4 (44%)	5 (56%)
LRCHL	8 (24%)	2 (25%)	6 (75%)

*M* male, *F* female, *CR* complete remission, *PR* partial remission, *PD* progressive disease. Histological subtype: *NSCHL* nodular sclerosis classic Hodgkin lymphoma, *MCCHL* mixed cellularity classic Hodgkin lymphoma, *LRCHL* lymphocyte rich classic Hodgkin lymphoma.

Histological diagnosis was nodular sclerosis CHL in 17 patients, mixed cellularity CHL in 9 patients, and lymphocyte-rich CHL in 8. Positive immunohistochemical reaction for TIGIT was observed in 19/34 patients (56%), of which 11 (58%) had an advanced clinical stage and 4 (21%) had bulky disease. Among the 15 TIGIT negative patients, 5 (33%) had an advanced stage and 11 (73%) achieved CR. Three patients that relapsed after response to treatment were all TIGIT+.

**Figure 1:**
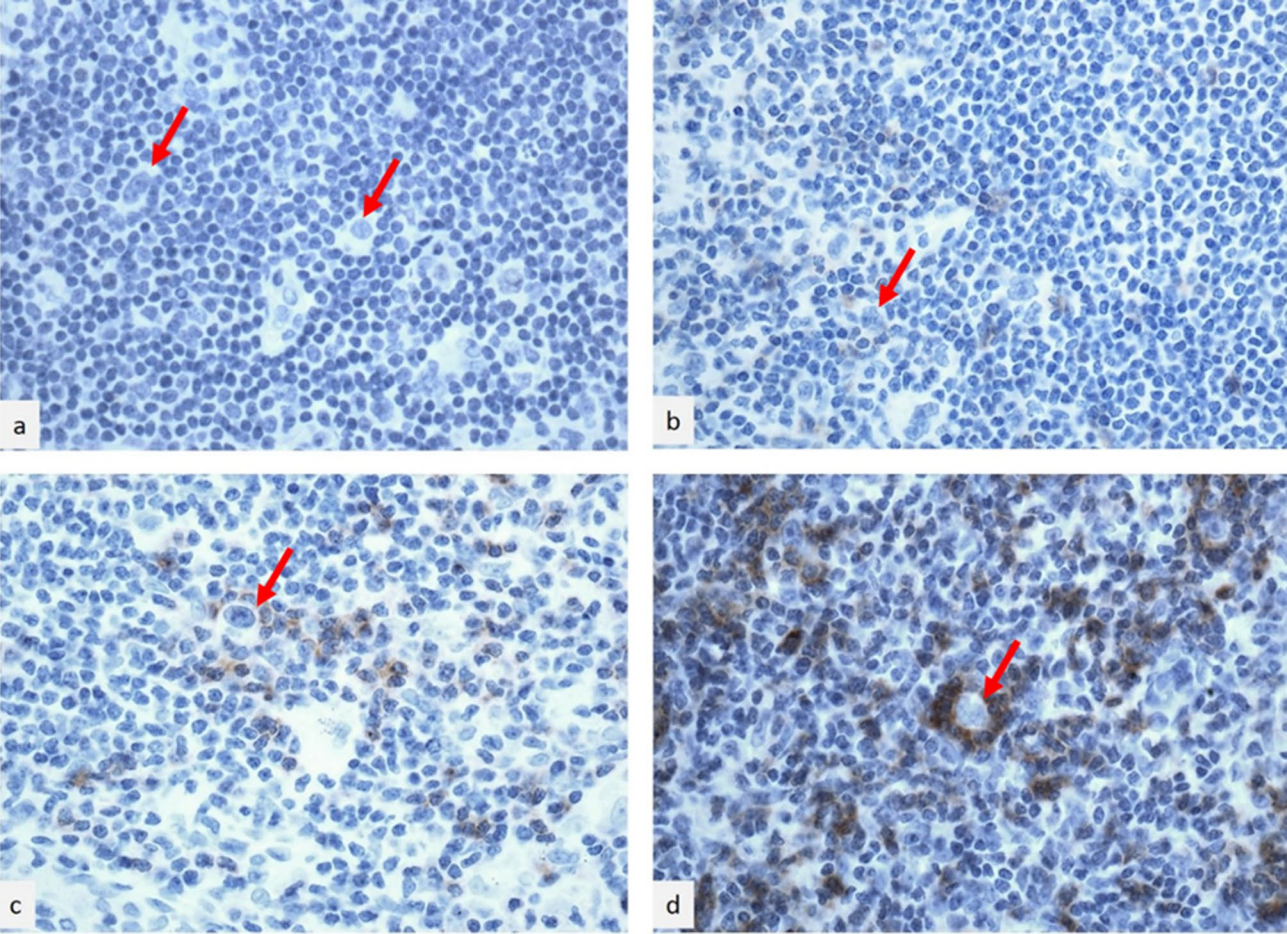
TIGIT immunohistochemistry in classical Hodgkin lymphoma. High power fields, hematoxylin counterstained. (**a**) Score 0: no evidence of lymphocytes with staining within the tumor environment; (**b**) score 1: sparse, faintly stained lymphocytes within the tumor environment, near the HRS cells; (**c**) score 2: presence of a discrete quote of lymphocytes with moderate membrane staining around the HRS cells; (**d**) score 3: evidence of a circle of lymphocytes with intense membrane staining surrounding the HRS cells. Red arrows indicate HRS cells.

The positive lymphocytes mainly corresponded to the CD3+, CD4+ T lymphocytes surrounding the HRS cells, although occasional CD8 cells may be also positive. The proposed scoring system was based on both intensity of reaction product and distribution of positive lymphocytes. Among 34 enrolled cases, 15 resulted negative for TIGIT immunohistochemistry on lymphocytes within the tumor environment and were classified score 0 (Fig. 1a). Ten cases showed a sparse, faintly stained non-tumoral lymphocytes within the tumor environment, near the HRS cells (Fig. 1b) and were classified as score 1. Five cases showed the presence of a discrete quote of non-tumoral lymphocytes with moderate membrane staining around the HRS cells (Fig. 1c) and were reported as score 2. Four cases demonstrated evidence of a circle of non-tumoral lymphocytes with intense membrane staining, surrounding the HRS cells (Fig. 1d), corresponding to the score 3.

There was complete agreement between the pathologists in assigning negativity (score 0) and maximum positivity (score 3). In the intermediate scores (score 1 and score 2) there was agreement in 9 of 15 cases (60%). The final scores for cases without concordance were assigned through a discussion between pathologists.

**Figure 2:**
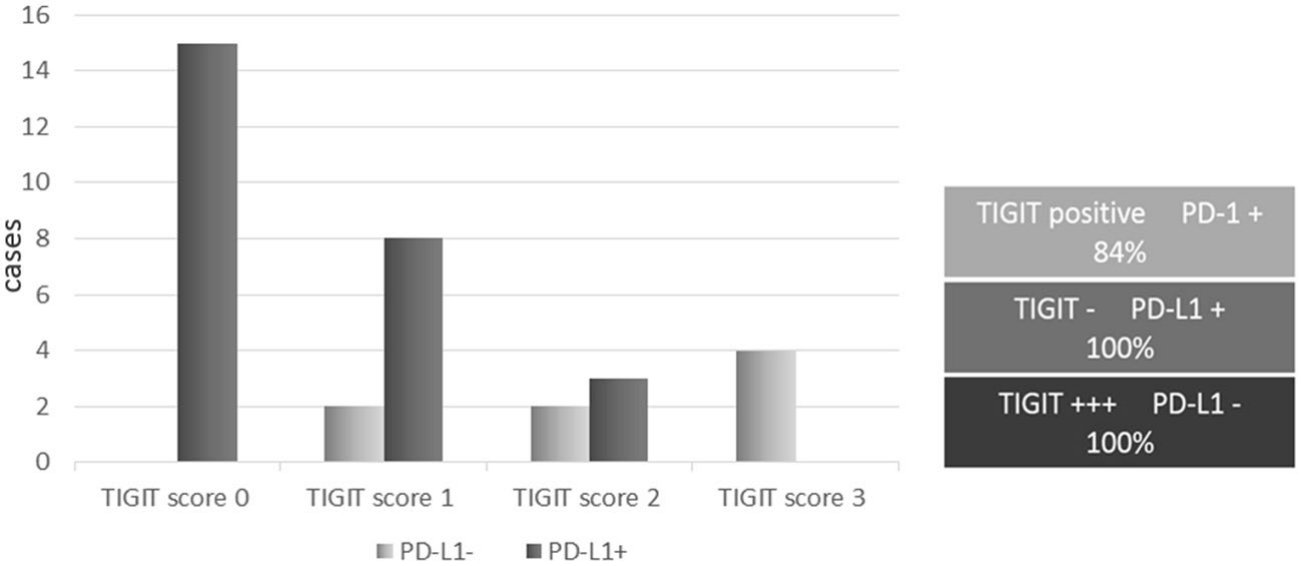
Left side: histogram shows the relationship between TIGIT and PD-L1 expression. PD-L1 expression decreases while TIGIT score increases. TIGIT negativity is always associated with PD-L1 positive reaction on HRS cells. The higher TIGIT expression is always associated with PD-L1 negativity on HRS cells. Right side: the scheme summarizes this result and underlines the close relationship between TIGIT expression and PD-1 positivity on peritumoral lymphocytes.

TIGIT immunoreaction was compared with immunohistochemistry for PD-1 and PD-L1 performed in each case on paired paraffin sections. Among 19 TIGIT positive cases, 16 (84%) showed PD-1 positive reaction on the same peritumoral lymphocytes while 3 resulted PD-1 negative. Among 15 TIGIT negative cases, only 4 (27%) were PD-1 positive and 11 (73%) were PD-1 negative (P = 0.001). PD-L1 correlation was performed by evaluating PD-L1 expression on HRS cells: among the 19 patients with TIGIT+ peritumoral lymphocytes, 11 (58%) were PD-L1+ on HRS cells, and all the 15 patients with negative TIGIT reaction in peritumoral lymphocytes were PD-L1+ in HRS cells (P = 0.0004). All TIGIT positive cases with the higher immunohistochemical expression (score 3) resulted negative for PD-L1 on HRS cells. These cases correspond to lymphocytic-rich CHL histological subtype (two cases) and to mixed cellularity CHL histological subtype (two cases). Figure 2 represents a visual image of how TIGIT immunohistochemical evidence correlates with PD-L1 expression (Fig. 2).

Positive controls stained correctly and negative controls failed to stain. Table 2 summarizes the histological subtype of the enrolled cases and the results of TIGIT, PD-1 and PD-L1 immunohistochemistry (Table 2).

**Table 2:** Histology and immunohistochemical results for TIGIT, PD-1 and PD-L1 expression.

Case	Histology*	TIGIT**	PD-1***	PD-L1****
1	NSCHL	1	+	+
2	MCCHL	0	−	+
3	NSCHL	0	−	+
4	LRCHL	3	+	−
5	NSCHL	2	+	−
6	MCCHL	0	−	+
7	NSCHL	1	+	−
8	NSCHL	0	−	+
9	NSCHL	0	−	+
10	MCCHL	2	+	−
11	LCCHL	2	+	+
12	MCCHL	3	+	−
13	LRCHL	1	+	+
14	NSCHL	1	+	+
15	LRCHL	3	+	−
16	MCCHL	1	+	+
17	NSCHL	1	−	+
18	NSCHL	2	+	+
19	MCCHL	3	+	−
20	NSCHL	2	+	+
21	LRCHL	1	−	−
22	NSCHL	0	−	+
23	NSCHL	0	−	+
24	MCCHL	0	−	+
25	NSCHL	0	+	+
26	NSCHL	0	+	+
27	LRCHL	1	+	+
28	NSCHL	0	+	+
29	NSCHL	1	−	+
30	NSCHL	0	−	+
31	LRCHL	0	−	+
32	MCCHL	0	+	+
33	LRCHL	0	−	+
34	MCCHL	1	+	+

*Histological subtype: *NSCHL* nodular sclerosis classic Hodgkin lymphoma, *MCCHL* mixed cellularity classic Hodgkin lymphoma, *LRCHL* lymphocyte rich classic Hodgkin lymphoma. **TIGIT expression was reported and graded from 0 to 3 using the proposed scoring system. ***PD-1 assessment was performed on lymphocytes surrounding HRS cells, normal lymphatic follicles were excluded from the count. ****PD-L1 positivity was defined as at least 5% positively staining HRS cells.

**Figure 3:**
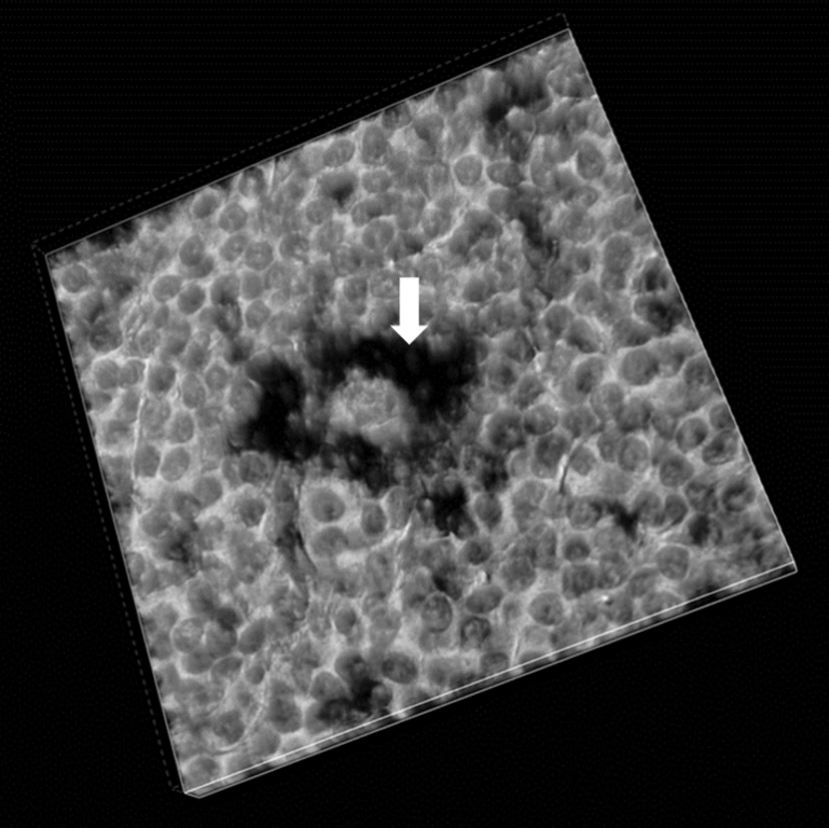
Confocal microscopy in light field from a case with intense TIGIT score. The black stained TIGIT+ lymphocytes (arrow) surround the HRS cell creating a fence against the immune environment.

We performed seriate sectioning and confocal microscopy to have a better image of the TIGIT shell around HRS cells. Confocal microscopy obtained from selected score 3 TIGIT positive cases, allowed us to obtain a three-dimensional reconstruction of the complex niche for HRS cells. The niche is constituted by TIGIT+ lymphocytes that completely surround the neoplastic HRS cell in an egg-shell shape (Fig. 3).

## Discussion

Our data confirm that TIGIT is frequently expressed in T lymphocytes within the tumor microenvironment of CHL^8^ and TIGIT expression seems to be related to CD4+ activated T lymphocytes surrounding the malignant HRS cells. The immune-morphologically well-known rosettes of T lymphocytes surrounding the neoplastic cells^10^, demonstrated by the three-dimensional reconstruction using a confocal microscopy, reveal to have actually an immune-suppressive functional role. Such fence may be responsible for producing ineffective immune-clearance of the malignant HRS cells and consequently resistance to treatment. Of relevance, HRS cells in cases without TIGIT expression in the surrounding T lymphocytes (score 0) were constantly PD-L1 positive. This result, compared with the complete negativity for PD-L1 of HRS cells entirely encircled by TIGIT+ lymphocytes (score 3), let us to speculate that the neoplastic cells use the immune-escaping mechanism mediated by TIGIT expression as an alternative system to the PD1/PD-L1 checkpoint. This mutually exclusive expression of TIGIT and PD-L1 observed when TIGIT is negative or highly expressed, may be a dynamic model that may varies over the time since PD-L1 was observed on HRS cells in cases with very low TIGIT expression.

As showed by the close interaction between malignant cells and activated TIGIT+ lymphocytes observed in the three-dimensional reconstruction of the niche, TIGIT expression may be induced in the T lymphocytes directly by HRS cells thorough presentation of TIGIT’s ligands such as PVR and CD112. This immunocheckpoints’ interaction has been hypothesized in other TIGIT positive neoplasms^11,12^.

On the clinical ground, the presence of TIGIT+ lymphocytes is slightly more frequent in patients with advanced stage disease: 58% in TIGIT+ cases vs 33% of TIGIT negative cases; TIGIT status seems do not correlate with response to treatment. Of relevance, the three patients that relapsed after response to treatment were all TIGIT+. This results needs for a more extensive analysis to confirm a possible role of TIGIT in HCL relapse. B symptoms were similarly distributed among TIGIT+ patients (53%) and TIGIT− patients (47%).

A point of interest is the co-expression of TIGIT and PD-1 in the peritumoral lymphocytes. Recently, a study comparing T cell subsets from tonsil and peripheral blood from patients with follicular lymphoma (FL) and healthy donors, demonstrated that TIGIT and PD-1 were the most frequently expressed co-inhibitory receptors^13^. Moreover, Wang et al. in patients with acute myeloid leukemia revealed that PD-1+ and TIGIT+CD8+ T cells are dysfunctional and associated with failure to achieve clinical remission^14^. Our data showed obtained by immunohistochemistry on seriate sectioning, showed co-expression of TIGIT and PD-1 on T lymphocytes in the Hodgkin microenvironment, resulting PD-1 positive 84% of the TIGIT positive cases, thus supporting a synergic co-inhibitory action in the HRS niche. Anyway, four cases TIGIT− showed PD-1 positive reaction on Tils as well, so indicating a little variability in this correlation, mainly linked to the NSCHL subtype (three out four cases, see Table 2). Recently Joe-Marc Chauvin reported that dual PD-1/TIGIT blockade potently increases tumor antigen-specific CD8+ T cell expansion and function in vitro and promotes tumor rejection in mouse tumor models. These findings support development of ongoing clinical trials with dual PD-1/TIGIT blockade in patients with cancer^15^.

Remaining in the field of hematological pathologies, Guillerey et al. demonstrated in TIGIT deficient mice with multiple myeloma, a decrease in monoclonal component associated with reduced tumor burden and prolonged survival. They observed also that in both mice and humans high levels of TIGIT expression on CD8+ T cells were associated with multiple myeloma progression, suggesting the development of TIGIT− blocking strategies in these patients^16^.

Reproducibility of TIGIT assessments was high with a 100% agreement between pathologists in assignment of negative and strong positive reaction. Among intermediate scores the agreement was 60%. This is a grey area, anyway we preferred to maintain score 1 and score 2 as separate entities because PD-L1 expression is different in these groups and future studies may find a clinical significance in such subdivision.

In conclusion, our preliminary results in Hodgkin lymphoma, demonstrated a mutually exclusive expression of TIGIT in peritumoral lymphocytes and PD-L1 in HRS cells, with an overlap in cases with low level of TIGIT reactivity. The new TIGIT scoring system is able to identify such modulation of these immunocheckpoints. These data encourage further studies evaluating the role of TIGIT as target for immunotherapies in CHL. The future challenge is to determine which patients are most suitable to receive these immunotherapies, and for whom combination therapy may be an appropriate treatment option.

## Materials and methods

In this study we enrolled 34 consecutive patients (17 males and 17 females) with CHL previously untreated, referred to Campus Bio-Medico University Hospital for diagnosis and treatment. Stage was defined according to the Ann Arbor staging system^17^. The study was approved by the Ethical Committee of the University Campus Biomedico of Rome (UCBM) (prot. 20.20 TS ComEt CBM). All enrolled patients signed an informed consent. Experiments were performed in full accordance with the principle of Good Clinical Practice (GCP) and the ethical principles contained in the current version of the Declaration of Helsinki.

Due to the limited number, we grouped the patients in the following two categories:

- Early stages, defined as those with Ann Arbor stage I and II;
- Advanced stages, defined as those with Ann Arbor stage ≥ III.

Formalin fixed-paraffin embedded lymph nodes of these patients were surgically removed at the time of the diagnosis. Final histological diagnosis included phenotypical characterization by immunohistochemical method with CD20 (Clone L26, Agilent), CD3 (Polyclonal Rabbit, Agilent), CD30 (Clone Ber-H2, Agilent), CD15 (Clone Carb-3, Agilent), CD4 (Clone 4B12, Agilent), CD45 (Clones 2B11 + PD7/26, Agilent), and Pax5 (Clone DAK-Pax5, Agilent) antibodies. Seriate paraffin sections from archive blocks of these cases were used for adjunctive immunohistochemistry with anti PD-1 (Clone NAT105, Cell Marque), PD-L1 (Clone QR1, UCS Diagnostic) and TIGIT (Clone TG1, Dianova) antibodies. Immunoreactions were achieved with automatized advanced staining system for immunohistochemistry with a controlled onboard environment (Omnis, Agilent), and visualized with diaminobenzidine. Negative controls were performed by omitting the primary antibody and positive controls were performed in agreement with manufacturer’s directions. Two pathologists with experience in hematologic disease (AC, AB) performed microscopic evaluation and the immunohistochemical assessment in blind. According to Menter et al. for PD-L1 assessment, only expression of PD-L1 on HRS cells was considered and PD-L1 positivity was defined as at least 5% positively staining tumor cells^18,19^. PD-1 assessment was performed on lymphocytes surrounding HRS cells, normal lymphatic follicles were excluded from the count. We considered PD-1 negative: cases with no staining on Tumor infiltrating lymphocytes (Tils), cases with PD-1 weak discontinuous membranous staining of Tils and cases with PD-1 staining in less than 2.8% of Tils^20^.

TIGIT immunohistochemical expression was evaluated with a score system ranging from 0 to 3, based on both intensity of the reaction and its distribution on the cell surface, allowing the reduction of the subjectivity of the method. The TIGIT score was assigned as follows (Fig. 1a–d):

- Score 0: no evidence of lymphocytes with membranous staining within the tumor environment (Fig. 1a);
- Score 1: sparse, faintly stained non-tumoral lymphocytes within the tumor environment, near the HRS cells (Fig. 1b);
- Score 2: presence of a discrete quote of non-tumoral lymphocytes with moderate membrane staining around the HRS cells (Fig. 1c);
- Score 3: evidence of a circle of non-tumoral lymphocytes with intense membrane staining surrounding the HRS cells (Fig. 1d).

The pathologists were in blind about clinical data and observed the immunostaining individually, with a stringent use of the scoring criteria. Selected cases were analyzed by for confocal microscopy (Nikon A1R+) with transmitted light detector in differential interference contrast (DIC), using primary TIGIT antibody and peroxidase development.

For the statistical analysis, data were analyzed by Statistical Package of Social Sciences software (SPSS, version 17.0, Chicago, USA). The comparison between the two patient cohorts was performed by using χ^2^ test and analysis of variance for categorical and quantitative variables, respectively. Two-sided P-values below 0.05 were considered statistically significant.

## Abbreviations

CHL: Classic Hodgkin lymphoma
TIGIT: T cell Ig and ITIM domains
HRS: Hodgkin Reed Sternberg
PD-1: Programmed death-1
PD-L1: Programmed death-ligand 1
TILs: Tumor infiltrating lymphocytes
T reg: Regulatory T cell
PVR: Poliovirus receptor
IgV: Immunoglobulin variable-set
ITIM: Immunoreceptor tyrosine-based inhibitory motif
ITT: Immunoglobulin tyrosine tail
PVRL2: Poliovirus receptor-related 2
ABVD: Adriamycin, bleomycin, vinblastine, and dacarbazine
CR: Complete remission
PR: Partial remission
PD: Progressive disease
NSCHL: Nodular sclerosis classic Hodgkin lymphoma
MCCHL: Mixed cellularity classic Hodgkin lymphoma
LRCHL: Lymphocyte rich classic Hodgkin lymphoma
AML: Acute myeloid leukemia
MM: Multiple myeloma
DIC: Differential interference contrast
SPSS: Statistical Package of Social Sciences
